# Physiological and molecular characterization of drought responses and identification of candidate tolerance genes in cassava

**DOI:** 10.1093/aobpla/plt007

**Published:** 2013-02-07

**Authors:** Laban F. Turyagyenda, Elizabeth B. Kizito, Morag Ferguson, Yona Baguma, Morris Agaba, Jagger J. W. Harvey, David S. O. Osiru

**Affiliations:** 1 Makerere University-Uganda, PO Box 7062, Kampala, Uganda; 2 National Agriculture Research Organization (NARO)-Uganda, PO Box 295, Entebbe, Uganda; 3 International Institute of Tropical Agriculture (IITA), c/o International Livestock Research Institute (ILRI), PO Box 30709, Nairobi 00100,Kenya; 4 The Nelson Mandela Institute of Science and Technology, PO Box 447, Arusha, Tanzania; 5 Biosciences Eastern and Central Africa–International Livestock Research Institute (BecA–ILRI) Hub, PO Box 30709, Nairobi 00100, Kenya

**Keywords:** Cassava, drought avoidance, drought tolerance, gene expression, osmotic adjustment, oxidative stress, real-time PCR

## Abstract

Cassava is an important root crop to resource-poor farmers in marginal areas, where its production faces drought stress constraints. Given the difficulties associated with cassava breeding, a molecular understanding of drought tolerance in cassava will help in the identification of markers for use in marker-assisted selection and genes for transgenic improvement of drought tolerance. This study was carried out to identify candidate drought-tolerance genes and expression-based markers of drought stress in cassava. One drought-tolerant (improved variety) and one drought-susceptible (farmer-preferred) cassava landrace were grown in the glasshouse under well-watered and water-stressed conditions. Their morphological, physiological and molecular responses to drought were characterized. Morphological and physiological measurements indicate that the tolerance of the improved variety is based on drought avoidance, through reduction of water loss via partial stomatal closure. Ten genes that have previously been biologically validated as conferring or being associated with drought tolerance in other plant species were confirmed as being drought responsive in cassava. Four genes (*MeALDH*, *MeZFP*, *MeMSD* and *MeRD28*) were identified as candidate cassava drought-tolerance genes, as they were exclusively up-regulated in the drought-tolerant genotype to comparable levels known to confer drought tolerance in other species. Based on these genes, we hypothesize that the basis of the tolerance at the cellular level is probably through mitigation of the oxidative burst and osmotic adjustment. This study provides an initial characterization of the molecular response of cassava to drought stress resembling field conditions. The drought-responsive genes can now be used as expression-based markers of drought stress tolerance in cassava, and the candidate tolerance genes tested in the context of breeding (as possible quantitative trait loci) and engineering drought tolerance in transgenics.

## Abstract (precis)

While the physiological basis of cassava drought tolerance has been characterized, evaluation of the molecular responses to drought stress remains largely unexplored. This study provides an initial characterization of the molecular response of cassava to drought stress resembling field conditions. The candidate drought tolerance genes in cassava identified in this study can be used as expression-based markers of drought tolerance in cassava or be tested in the context of breeding and engineering drought tolerance in transgenics.

## Introduction

Improvement and expanded adoption of crops suited to growth with limited water resources on marginal lands is critical to ensuring food security, given the limited arable land and population growth, further compounded by the effects of climate change. In sub-Saharan Africa (SSA) and throughout much of the tropics and sub-tropics, the development and use of crop varieties with high water-use efficiency is particularly important for marginal areas with poor soils, unreliable rainfall and where irrigation is unavailable or unaffordable for resource-poor farmers. In this respect, cassava deserves particular attention because of its status and further potential as both a food security and a cash crop for most households living in marginal areas of the tropics and sub-tropics. In the tropics, cassava ranks third as a source of calories, and is typically grown by resource-poor smallholder farmers on marginal lands. A naturally drought-tolerant (DT) crop, it provides a critical staple to many of these populations vulnerable to food insecurity.

It has been estimated that moisture or drought stress is the most adverse crop environmental stress, accounting for over 70 % of potential agriculture yield losses worldwide (Boyer 1982). Drought is brought about by a shortage of rain or by a large variation in the amount of rainfall, and is the major abiotic stress limiting crop productivity worldwide (Saini and Westgate 2000). In Africa, the cassava growth cycle is typically interrupted by 3–6 months of drought, influencing various plant physiological processes resulting in depressed growth, development and economic yield (Pardales *et al.* 2001; Bakayoko *et al.* 2009). In general, cassava can withstand significant periods of drought stress. However, there is a range of drought-tolerance levels in available germplasm, and its growth and productivity in marginal areas are constrained by severe drought stress, especially during the earlier stages of growth (Pardales *et al*. 2001; Okogbenin *et al*. 2003; Bergantin *et al*. 2004; Perez *et al.* 2011). Development of cassava varieties with farmer-preferred traits and increased drought tolerance will allow its expanded cultivation and elevated yields in marginal areas.

Given the inherent challenges with cassava breeding, an understanding of the molecular basis of cassava drought responses and tolerance can help greatly in the development of appropriate varieties (Valliyodan and Nguyen 2006; El-Sharkawy 2007). Conventional breeding has been hindered by cassava's high heterozygosity, genotype by environment (G × E) interaction, long life cycle (Hahn *et al.* 1989; Fregene *et al.* 2001; Ceballos *et al.* 2004) and limited seed production, while molecular breeding is hindered by limited information on genomic regions and genes associated with drought tolerance in cassava. Efforts to improve cassava's water-use efficiency through conventional breeding have been limited in many parts of the world, including much of SSA. Breeding programmes in Latin America have successfully identified germplasm with increased levels of drought tolerance, with 2–3 times the yield of typical cassava genotypes in semi-arid conditions (El-Sharkawy 2007). A range of cassava drought-tolerance levels has also been characterized in West Africa (Okogbenin *et al.* 2003). Efforts in are now under way in eastern Africa to begin breeding for DT cassava.

Molecular breeding has already formed the basis of significant progress for other cassava traits. For instance, molecular markers tightly associated with the cassava mosaic disease (CMD) resistance gene *CMD2* have been used in marker assistance breeding for CMD resistance (Akano *et al.* 2002). Breeding for tolerance to cassava postharvest physiological deterioration has also been reported (Morante *et al.* 2010). However, little progress has been made with respect to the development of DT cassava varieties. An understanding of the molecular response and basis of drought tolerance in cassava would significantly accelerate the production of DT varieties with farmer-preferred traits, through molecular breeding or genetic transformation, both of which have been successful in the development of DT plants. Utsumi *et al.* (2012) identified cassava genes responsive to drought treatment that consisted of wilting *in vitro* plantlets on a plastic plate for 1h under light. Further studies are required using drought stress methods more closely resembling drought stress conditions in the field in order to more confidently identify candidates appropriate for use in efforts to improve cassava drought tolerance.

Plant tolerance to drought stress is a complex trait with several interacting layers of molecular and physiological responses. Drought stress responses and tolerance genes have been well characterized in a number of plant species (Farooq *et al.* 2009; Gong *et al.* 2010), lending insight into the general pathways involved and potential tolerance mechanisms and genes in other species. Plant resistance to drought stress can be achieved through escape (e.g. early flowering time in drier environments), avoidance (e.g. transpiration control by stomata and development of extensive root systems), phenotypic flexibility, water conservation in tissues, antioxidant defences, plant growth regulation by hormones and osmotic adjustment (Farooq *et al.* 2009). Drought stress induces accumulation of metabolites and drought-related proteins (Ramachandra-Reddy *et al*. 2004). At the molecular level, the response to drought stress is a multi-genic trait. Through high-throughput microarray and real-time polymerase chain reaction (PCR) studies, a number of genes that respond to drought stress at the transcriptional level have been reported (Seki *et al.* 2002; Shinozaki and Yamaguchi-Shinozaki 2007; Talame *et al.* 2007; Guo *et al.* 2009). Some of these genes have been validated biologically and have been found to protect plants from desiccation through stress perception, signal transduction, transcriptional regulatory networks in cellular responses or tolerance to dehydration (Wang *et al.* 2005; Umezawa *et al.* 2006). Drought-stress-induced regulatory and functional genes have been used to increase drought tolerance through genetic engineering; for example, *Vigna aconitifolia pyrroline-5-carboxylate synthetase (P5CS)* has been used to engineer DT rice, and *manganese superoxide dismutase (MnSOD)* for DT alfalfa (McKersie *et al.* 1996; Zhu *et al*. 1998).

Cassava drought stress has been characterized physiologically and morphologically (reviewed by El-Sharkawy 2004; Setter and Fregene 2007; Okogbenin *et al*. 2011), and at the molecular level under conditions requiring further investigation to ensure their relevance to the context of field drought stress (Utsumi *et al.* 2012). Ecophysiologically, mechanisms of drought tolerance in cassava have been identified such as avoidance, through partial stomatal closure to reduce transpiration, development of extensive root systems and proportionally strategic reductions in leaf canopy (El-Sharkawy 2004, 2007); however, in some studies greater leaf retention has been correlated with drought tolerance (Lenis *et al.* 2006), so the relationship between leaf retention and drought tolerance depends on the genotype and probably on environmental factors (e.g. severity of drought). While a limited number of molecular studies have sequenced normalized expressed sequence tag libraries from cassava under drought stress (Lokko *et al.* 2007), no molecular studies have been conducted that quantify gene expression in single or contrasting cassava genotypes under conditions resembling those in the field, which would enable the identification of both drought-responsive and candidate drought-tolerance genes most relevant to cassava drought improvement efforts.

This study confirmed the DT and drought-susceptible (DS) status of improved and farmer-preferred cassava varieties, respectively, which are now part of the germplasm being integrated into the breeding programme at the National Crops Resources Research Institute (NACRRI) in Uganda to develop DT cassava with other farmer-preferred traits. The morphological and physiological responses of the two genotypes to drought stress were assessed. The relative expression levels of genes previously demonstrated to be functionally involved in, or associated with, drought stress responses in other species were also analysed. This study provides a general characterization of drought responses in cassava, yielding expression-based markers and candidate drought-tolerance genes for ongoing cassava improvement efforts. A molecular understanding of the drought responses of this DT species can also provide insights for increasing the drought tolerance of more drought-sensitive species.

## Methods

### Genotypes and treatments

Two cassava genotypes, DT MH96/0686 and DS Nyalanda, were used in this study. MH96/0686 is an improved cassava variety obtained from the cassava breeding programme at NACRRI, Uganda, while Nyalanda is a landrace obtained from farmers' fields in the Masindi district in western Uganda (1°40′28″ N, 31°42′54″ E). Previous field studies of 53 cassava genotypes in Uganda indicated that MH96/0686 was tolerant to drought and had a high harvest index, dry matter content, starch content, root yield and leaf retention under water stress compared with other genotypes, while Nyalanda was among the genotypes adversely affected by water stress and was significantly different from MH96/0686 in these phenotypes under drought stress (Turyagyenda *et al*. 2013). Based on the field data, these two genotypes were selected for detailed gene expression studies that were conducted in a glasshouse. The glasshouse conditions during the day were set at 25–30 °C (Alfredo and Setter 2004) with night-time temperatures typically ranging between 15 and 20 °C, and humidity typically at ∼50–80 %.

Cassava cuttings (30 cm in length) of these two genotypes were grown in 20-L plastic buckets in a randomized complete block design (RCBD) replicated three times. Before planting, each bucket was filled with 20 kg of sterilized soil (forest soil : river sand : ballast at 4 : 2 : 1 (v/v/v) respectively). One plant cutting was placed vertically in the soil in the middle of each bucket. To identify the effect of water stress on gene expression, plants were exposed to water stress by reducing soil moisture content (SMC). A similar number of plants per genotype remained watered at field capacity to act as a control. Three plants per genotype were included for each replication for each treatment. Before the application of treatments, all plants were watered with 1 L of water every 2 days until 60 days after planting (DAP). After 60 DAP, plants in the stress treatment were subjected to gradual drought stress conditions for a total of 10 days by withholding water to an SMC of ∼50 % for 5 days and then to ∼25 % for 5 days; gradual moisture stress was applied to mimic natural field drought conditions. Control (well-watered) plants were maintained at an SMC of ≥75 % by applying 1 L of water every 24 h. The SMC was monitored daily with a portable moisture meter (Delta Systems, UK).

### Morphological and physiological drought stress measurements

During the water stress treatment period, physiological and morphological drought-stress-related traits were measured, including: leaf retention and plant height; and stomatal conductance, leaf relative water content (RWC) and leaf wilting. These measurements were collected from three plants per genotype from each replication for each treatment after 10 days of drought stress, just before leaf collection for gene expression analysis. Leaf wilting was scored on a scale of 1–3 modified from Bettina *et al.* (2007) (1 = no wilting; 2 = minimal wilting, where the plant showed leaf wilting only during hot hours and from which the leaves recovered; and 3 = severe wilting, where wilting leaves did not recover from wilting). For easy scoring, Bettina *et al.* (2007) scales 2 and 3 were combined to indicate minimal wilting, and 4 and 5 to indicate severe wilting. The stress treatment did not go as far as killing plants and therefore level 6 (death) (Bettina *et al.* 2007) was eliminated. The method of visual scoring of wilting is flexible as long as specific wilting categories are defined appropriately (Bettina *et al.* 2007). Stomatal conductance was measured on the third fully expanded leaf with an AP4 Porometer (Delta-T Devices, UK), while RWC was estimated on the fourth fully expanded leaf by following the procedure of Degenkolbe *et al.* (2009). Leaf retention was estimated as the percentage of the portion of stem height with leaves, and plant height was measured with a tape measure.

### Leaf harvesting and RNA extraction

After 10 days of stress, the third fully expanded leaf was collected separately from three plants per genotype from each replication for each treatment. Leaf samples were collected between 12:00 and 12:30 p.m. To avoid taking material from the elongation zone at the base of the leaf blade or senescent tissue at the tip of the leaves, leaf samples were harvested from the middle section of the blades of fully expanded green leaves (Degenkolbe *et al.* 2009). The leaf samples were collected in Eppendorf tubes, immediately put in liquid nitrogen and stored at −80 °C until RNA extraction. Total RNA was extracted from the leaf samples (three biological replicates per genotype per treatment) using Concert Plant RNA Reagent (catalogue number 12322-012; Invitrogen) following the manufacturer's protocol. The concentration of RNA from each sample was determined by UV spectrophotometry at *A*_260_ using NanoDrop ND-1000 (NanoDrop Technologies, USA), while the integrity of total RNA was analysed by both Nanodrop (A_260_/A_280_) and 1.5 % 1× Tris-borate-EDTA (TBE) agarose gel electrophoresis (visualized with ethidium bromide; EtBr) after denaturation in 1×FDE (90 % v/v formamide, 1× TBE buffer, 0.5 % w/v bromophenol blue, 25 mM EDTA) at 65 °C for 5min and snap cooling on ice.

DNA contamination was removed using RNase-free DNaseI (Fermentas cat. no. EN0521) following the manufacturer's protocol. Two reverse transcription reactions (two technical replications) were prepared from each biological replicate. One microgram of total RNA was converted to cDNA by the GoScript reverse transcriptase system (Promega, USA) following the manufacturer's instructions. Briefly, 1 µg of total RNA was mixed with 10 pmol of random hexamer oligonucleotides to a final volume of 10 µL and the mixture incubated at 75 °C for 5 min. Then 1 µL of GoScript reverse transcriptase (Promega), 0.5 µL of RNasin RNase inhibitor (Promega), 4 µL of 5× PCR buffer, 1 µL of 10 mM dNTP and 1.2 µL (25 mM) of MgCl_2_ were added to the mixture on ice. Ribonuclease-free water was added to a final volume of 20 µL and the mixture incubated at an annealing temperature of 25 °C for 5 min. Extension was carried out at 42 °C for 60 min and the reaction was inactivated at 70 °C for 15 min. Two control reactions were included for each sample throughout this process: one without reverse transcriptase and one without RNA template.

### Gene identification and PCR optimization

A literature survey was conducted to identify genes that have been functionally confirmed to confer drought tolerance in at least one plant species. The National Centre for Biotechnology Information (NCBI) database (**http://www.ncbi.nlm.nih.gov/**, accessed in June 2010) cDNA sequences of these genes were used as queries to identify cassava homologues through BLAST searches of the cassava genome database (**http://www.phytozome.net**, accessed in June 2010). This resulted in the identification of cassava gene homologues in cassava. When the query sequence was highly similar to several cassava genes (i.e. in a gene family), a multiple alignment of the highly similar cassava homologues was conducted with EBI clustalw2 tool (**http://www.ebi.ac.uk/Tools/clustalw2/index.html**, accessed in June 2010). Only homologues with high scores, percentage nucleotide identity to the gene of interest and high coverage of the query sequence were considered for clustering. Primers suitable for quantitative reverse transcription-PCR (qRT-PCR) (amplifying 200– to 350–bp products) and specific to the gene of interest (i.e. to a full-length coding sequence exhibiting the highest similarity to the query sequence; through maximum specificity at the 3′ end of the primer) were designed to amplify the identified cassava homologues.

The primers were optimized for target gene specificity with endpoint PCR using cassava cDNA. The endpoint PCR products were visualized by electrophoresis on a 2 % agarose 1× TBE gel, and visualized with EtBr. The PCR products for each primer pair were sequenced and compared with target genes (Table 2 ‘cassava homologues’ column) to confirm that the correct cassava genes were being amplified. All the designed primers (100 %) amplified cDNA to the expected product with a single band at an annealing temperature of 55 °C on an endpoint PCR system and thus this temperature was selected as the appropriate annealing temperature for qRT-PCR.

### Quantitative PCR

Quantitative RT-PCR was performed on a 7500HT standard Real-Time PCR system (ABI-PRISM^®^, USA) using SYBR Green JumpStart TaqReadyMix (Sigma, USA). The qRT-PCR was run on three biological replicates for each treatment for each genotype. Duplicate reactions were run for every biological replicate. The 20-µL reaction volume consisted of the following: 10 µL of 2× SYBR Green I ready mix, 0.02 µL of passive reference dye, 1 µL (10 pmoL) each of the forward (F) and reverse (R) gene-specific primers, 2 µL of template cDNA (50 ng) and 5.98 µL of distilled, deionized water (ddH_2_O). The PCR conditions were as follows: initial denaturation at 94 °C for 2 min; 40 cycles of denaturation at 94 °C for 15 s, annealing at 55 °C for 1 min and extension at 60 °C for 30 s. The dissociation curve analysis was carried out at the default setting of the 7500HT Real-Time PCR system to confirm the specificity of each reaction. A subset of the amplification products was also run on a 1× TBE agarose gel, stained with EtBr to ensure that each primer pair had one specific product.

The qRT-PCR reactions were normalized with the cassava *actin* gene (primers F: 5′-TGCAGACCGTATGAGCAAG-3′; R: 5′-CACCCTTGGAAATCCACATC-3′) as reference for all comparisons (Guo *et al.* 2009; Yang *et al*. 2011). The reference gene was expressed at similar levels in both well-watered and water-stressed treatments for both genotypes. The amplification efficiency of primers was determined by performing qRT-PCR on 1 : 2; 1 : 4, 1 : 8; 1 : 16; 1 : 32 and 1 : 64 dilutions of cDNA pooled from all experimental samples. All primer pairs amplified the genes with approximately the same efficiency as that of the reference gene *actin* and ranged from 1.96 to 2.01. The ΔΔCT method of relative gene quantification was used to conduct the various comparisons of relative gene expression from the qRT-PCR data, using the Relative Expression Software Tool (REST) (Pfaffl *et al.* 2002). A gene is significantly up-regulated or down-regulated when its expression in a treatment is higher or lower than that in a calibrator (standard/baseline), respectively, and when the *t*-test statistic is lower than 0.05 (at 95 % significant level). The expression in a calibrator is taken as unit (one), expression more than one is up-regulation and expression less than one is down-regulation. The *t*-statistic will show whether the up-regulation or down-regulation is significant or non-significant (NS).

## Results

### Morphological and physiological drought stress characteristics

**Fig. 1:**
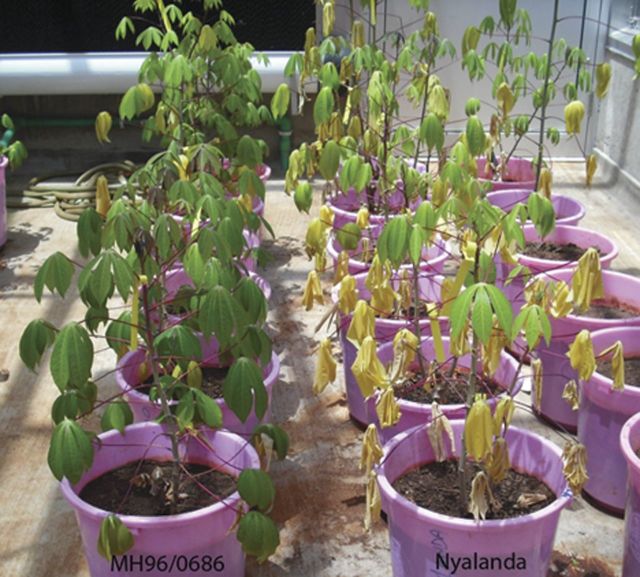
**Effect of drought stress on improved MH96/0686 cassava genotype and landrace Nyalanda.** Stress treatment was gradually given to the plants 60 days after planting. Moisture stress was gradually applied to mimic natural field drought conditions. Improved MH96/0686 and farmer preferred landrace Nyalanda were differentially affected by drought stress conditions. After 10 days of gradual application of drought stress, MH96/0686 was less affected by water stress than Nyalanda, which exhibited marked wilting and other drought stress symptoms.

At T0 (day 1/first day of drought treatment), the two genotypes were healthy and exhibited no readily observable symptoms of drought stress. After 10 days of reduced SMC, the two genotypes exhibited different levels of drought stress: the DS genotype Nyalanda showed severe wilting symptoms on the leaves, including mild chlorosis of upper leaves and senescence of many of the lower leaves, compared with more limited stress signs in the DT genotype MH96/0686 (Fig. 1). In order to assess the drought stress response in leaves that were at the onset of visible stress in the DS genotype at the 10-day time point, RWC and stomatal conductance were gathered from leaves just beginning to exhibit wilting symptoms. The mean SMC in stressed plants was significantly lower (28.80 ± 1.08) than that in well-watered plants (83.00 ± 2.45). Nyalanda lost 19 % of its leaves through shedding under stress while MH96/0686 retained almost all its leaves (over 99 %), (i.e. the ‘stay green’ trait) (Table 1 and Fig. 1). Nyalanda leaves were permanently wilted, compared with minimal leaf wilting of MH96/0686 genotype leaves that was limited to hot hours (∼1200–1500 h), after which the leaves recovered from wilting. In MH96/0686, stomatal conductance was more than two times lower than in Nyalanda under stress.

**Table 1:** **Physiological and morphological responses of the two genotypes after 10 days of moisture stress.** Physiological and morphological drought-stress-related traits, measured on three plants per replication for each treatment (stressed and control) after 10 days of water stress (just before leaf sample collection for qRT-PCR). All values shown are mean values at *P* ≤ 0.05. ns, not significant; **significant at *P* ≤ 0.05; SBG, significance between genotypes (columns); significance between treatments is shown in the rows.

Cultivar	Conductance (mmol m^−2^ s^−1^)		Leaf retention (%)		Relative water content (%)		Number of leaves		Plant height (cm) ns	
	Control	Stressed	Control	Stressed	Control	Stressed	Control	Stressed	Control	Stressed
MH96/0686	350.0 ± 37.21	168.9 ± 200**	76.88 ± 3.65	76.33 ± 2.33ns	97.3 ± 1.12	95.4 ± 2.92ns	26.78 ± 1.27	24.89 ± 1.50 ns	52.22 ± 2.86	52.11 ± 2.64 ns
Nyalanda	492.5 ± 43.0	355 ± 21.2**	63.33 ± 4.22	51.25 ± 2.48**	94.95 ± 1.37	81.3 ± 2.92ns	21.50 ± 1.56	13.37 ± 1.59**	48.67 ± 3.51	47.50 ± 2.80 ns
SBG	**	**	**	**	ns	**	**	**	ns	ns

### Cassava drought stress gene PCR assay validation

Of the 10 genes previously confirmed to have a functional role in drought stress that were selected for this study (Table 2), seven were previously reported from studies in *Arabidopsis thaliana*, one in *V. aconitifolia*, one in *Pisum sativum* and one in *Oryza sativa*. All designed primers (Table 2) amplified cDNA with a single band at the expected size and of the expected sequence, based on the cassava database sequences from which the primers were designed (data not shown).

**Table 2:** **The primers designed for the 10 genes used in gene expression analysis.** cDNA sequences of genes that confer drought tolerance in at least one plant species were used as queries to identify cassava homologues through BLAST searches of the cassava genome database. The cassava homologues were then used to manually design primers suitable for qRT-PCR (amplifying 200- to 350-bp products).

Gene name	Accession	Mode of action	Cassava homologues (target genes)^a^	e-value (two species' genes)	Primer ID	Primer sequence (5′–3′)	Length	Expected size (bp)
*Oryza sativa* Japonica Group zinc finger protein ZFP252	AY219847	Osmotic adjustment through proline and sugars	cassava4.1_014662m.g (*MeZFP*^a^)	0	ZFP1F	CTC TAT TCT CAG CGC ACA TTC C	22	245
					ZFP1R	AGC ATA ACG AGG CAG AGA GC	20	
*Arabidopsis thaliana* amino acid transporter family II protein	NM_129684	Likely role in osmotic adjustment	cassava4.1_007924m.g (*MeATTF*)	2.7e-36	ATTF1F	GTG GAA CTT TCT CCT CTC AGC A	22	300
					ATTF1R	GCG TTA AAC TAC ATC CAT GGG C	22	
*Arabidopsis thaliana* ALDH7B4	NM_104287	Antioxidant/ROS scavenging	cassava4.1_014540m.g (*MeALDH*)	5.9e-43	ALDH1F	GGA TGG AAT GCA TGC ATT GCA CTG	24	263
					ALDH1R	CTG ATT CAC TGT TTG TTG CAC CAT C	25
*Pisum sativum manganese superoxide dismutase*	U30841	Antioxidant/ROS scavenging/detoxication	cassava4.1_015272m.g (*MeMSD*)	4.8e-40	MSD1F	ATG AAT GCA GAA GGT GCT GCA	21	269
					MSD1R	GAA GGG CAT TCT TTG GCA TAC	21	
*Arabidopsis thaliana* GER3 (GERMIN 3)	NM_122070	Regulation of plant growth	cassava4.1_016243m.g (*MeGE3*)	2.4e-51	GE31F	CGC TTG CAA GAA ACC TGC AG	20	254
					GE31R	TGA ACC CAG CAC AGA TAG AC	20	
*Arabidopsis thaliana* GBF3 (G-BOX BINDING FACTOR 3);	NM 180118	Transcription factor and regulates alcohol dehydrogenase (Adh) via ABA	cassava4.1_008459m.g (*MeGBF3*)	1.9e-18	GBF32F	TGC ATC AAC TGT TGG GTG CG	20	244
					GBF32R	ACC CAG AGC CAT GAG AAG GCT	21	
*Arabidopsis thaliana* 14-3-3 protein GF14 lambda (GRF6)	AF145298	Signalling factor/Delay leaf senescence (stay green trait)	cassava4.1_014556m.g (*MeGF14*)	1.3e-104	GF141F	AGC ACG CTT CTC TCT CTC TC	20	261
					GF141R	AGG AAA CGA TCC TCC AAG CG	20	
*Arabidopsis thaliana* RD28	NM_129274	Turgor responsive/transport of small molecules across membranes	cassava4.1_013192m.g (*MeRD28*)	7.6e-64	RD282F	TGC ACT GCT GGT ATC TCA GG	20	237
					RD282R	GAT CTC AGC TCC CAA TCC AG	20	
*Arabidopsis thaliana* MYC2	NM_102998	Transcription factor and regulates ABA-dependent RD22 and ADH1	cassava4.1_002918m.g (*MeMYC2*)	1.1e-40	MYC21F	AGC GTC TCC AGA CCT TGA TC	20	233
					MYC21R	AGT GGG ACC TGA GAT CAG C	19	
*Vigna aconitifolia pyrroline-5-carboxylate synthetase*	M92276.1	Osmotic adjustment	cassava4.1_002381m.g (*MeP5CS*)	1.4e-78	VAP1F	AGA CGT TAA GCG TAT CGT TG	20	332
					VAP1R	CAA GAA GTT GAG CTG ATG TC	20	

^a^The cassava homologues in parentheses were assigned gene names starting with ‘Me’ for *Manihot esculenta*.

### Gene expression analysis

**Fig. 2:**
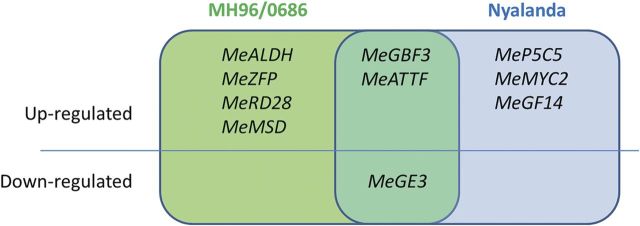
**Gene expression changes induced by drought stress in the two genotypes.** The relative changes in expression of each gene between the DT and DS genotypes are summarized according to the analysis of relative expression changes under drought stress compared with well-watered control conditions.

Several comparative analyses were conducted to determine the dynamics of the drought response gene expression changes in the two genotypes. In all analyses, the drought response gene expression levels were first normalized to the control gene (*actin*). The first question addressed was whether the selected genes are in fact drought responsive in cassava. For this, the expression of each of the 10 genes was compared between well-watered controls and drought-stress-treated plants within each of the two genotypes (Table 3). The expression of each of the 10 genes responded to water stress in one or both genotypes: nine of the genes were up-regulated in one or both genotypes, and one gene (*MeGE3*) was down-regulated in both. In the susceptible genotype Nyalanda, six genes were differentially expressed across treatments, five of which were up-regulated (*MeATTF*, *MeGBF3*, *MeGF14*, *MeP5CS* and *MeMYC2*) and one of which was down-regulated (*MeGE3*); four genes (*MeALDH*, *MeMSD*, *MeRD28* and *MeZFP*) were not differentially expressed in response to water stress. In the tolerant genotype MH96/0686, seven genes were differentially expressed, of which six (*MeALDH*, *MeATTF*, *MeGBF3*, *MeMSD*, *MeRD28* and *MeZFP*) were significantly up-regulated and one (*MeGE3)* was down-regulated by drought stress; three genes (*MeGF14*, *MeMYC2* and *MeP5CS*) were not differentially expressed. All 10 genes responded to drought, with differences in which genes were responsive to drought in the two genotypes (Fig. 2).

**Table 3:** **Effect of water stress on mRNA levels, comparing stressed to control plants within a genotype.** Quantitative RT-PCR was performed for each identified gene on three biological replicates for each treatment (stress and control) for each genotype (MH96/0686 and Nyalanda). Duplicate reactions were run for every biological replicate. The qRT-PCR reactions were normalized with the cassava *actin* gene as a reference for all comparisons. The ΔΔCT method of relative gene quantification was used to make the various comparisons of relative gene expression from the qRT-PCR data, using REST. For each genotype, the control plants were used as a calibrator. A gene is significantly up-regulated or down-regulated when its expression in a treatment is higher than or lower than that in a calibrator (standard/baseline), respectively, and when the *t*-test statistic is lower than 0.05 (at 95 % significance level). The expression in a calibrator is taken as unity (one), expression of more than one is up-regulation and expression less than one is down-regulation. The *t*-statistic will show whether the up-regulation or down-regulation is significant or non-significant (NS).

Genotype	Gene	Expression	SE	95 % CI	Probability	Result
MH96/0686 Stressed against well watered	*MeALDH*	2.815	1.818–4.183	1.327–6.112	0.000	Up-regulated
	*MeATTF*	3.245	1.444–9.582	1.069–11.530	0.000	Up-regulated
	*MeGBF3*	3.241	2.221–5.467	1.688–9.982	0.000	Up-regulated
	*MeGE3*	0.317	0.181–0.647	0.097–0.988	0.006	Down-regulated
	*MeGF14*	1.303	0.963–1.768	0.844–2.344	0.095	NS
	*MeMYC2*	1.350	0.718–2.196	0.608–3.336	0.204	NS
	*MeMSD*	3.148	2.316–4.431	1.897-6.394	0.001	Up-regulated
	*MeRD28*	1.511	1.062–1.998	0.852–2.153	0.013	Up-regulated
	*MeP5CS*	1.425	0.686–3.784	0.384–4.745	0.301	NS
	*MeZFP*	4.043	2.869–5.828	2.164–8.014	0.000	Up-regulated
NYALANDA Stressed against well watered	*MeALDH*	2.160	1.003–5.120	0.464–7.983	0.056	NS
	*MeATTF*	2.671	1.902–3.812	1.393–4.954	0.001	Up-regulated
	*MeGBF3*	1.875	1.161–3.028	0.733–3.979	0.018	Up-regulated
	*MeGE3*	0.205	0.057–0.671	0.034–0.941	0.000	Down-regulated
	*MeGF14*	2.285	1.094–6.336	0.826–18.602	0.031	Up-regulated
	*MeMYC2*	2.201	1.391–3.371	0.976–4.183	0.006	Up-regulated
	*MeMSD*	1.506	0.983–2.255	0.815–4.129	0.063	NS
	*MeRD28*	1.128	0.994–1.286	0.875–1.381	0.059	NS
	*MeP5CS*	1.662	1.125–2.414	0.977–3.496	0.007	Up-regulated
	*MeZFP*	1.578	0.806–3.368	0.525–5.198	0.159	NS

CI, confidence interval at 95 %; expression, fold change in the expression of a gene in water stress relative to control treatment (*P* = 0.05).

Given that the expression of the 10 previously identified plant drought-tolerance genes is responsive to drought in cassava, the next question was whether their expression can provide insight into the basis of the relative drought tolerance and susceptibility of the two genotypes; genes expressed at higher levels in MH96/0686 compared with Nyalanda are candidates for conferring drought tolerance. For this, two comparisons between genotypes were made: relative expression levels under non-stressed conditions (basal expression levels) and relative expression levels under drought stress.

To determine the relative expression levels under non-stressed conditions, baseline expression levels were compared under well-watered conditions for the two genotypes (Table 4). Even in the absence of drought stress, three genes exhibit different expression levels in the two genotypes. *MeGF14* and *MeMYC2* are expressed at approximately twice the level in MH96/0686 (DT) compared with Nyalanda (DS). Alternatively, *MeMSD* is expressed at approximately half the level in DT compared with DS.

**Table 4:** **Comparison of the baseline gene expressions of DT MH96/0686 and DS Nyalanda.** Quantitative RT-PCR was performed for each identified gene on three biological replicates for well-watered plants (control) of each genotype (MH96/0686 and Nyalanda). Duplicate reactions were run for every biological replicate. The qRT-PCR reactions were normalized with the cassava *actin* gene as a reference for all comparisons. The ΔΔCT method of relative gene quantification was used to make the various comparisons of relative gene expression from the qRT-PCR data, using REST. During data collection, Nyalanda (DS landrace) was used as a calibrator. The DT genotype has several drought-tolerance genes whose levels are different from those in DS (under well-watered conditions). A gene is significantly up-regulated or down-regulated when its expression in a treatment is higher than or lower than that in a calibrator (standard/baseline), respectively, and when the *t*-test statistic is lower than 0.05 (at 95 % significant level). The expression in a calibrator is taken as unity (one), expression of more than one is up-regulation and expression less than one is down-regulation. The *t*-statistic will show whether the up-regulation or down-regulation is significant or non-significant (NS).

Gene	Expression (fold difference in baseline expression levels, DT vs. DS)	SE	95 % CI	Probability (*P* = 0.05)	Relative significant difference (DT vs. DS)
*MeALDH*	1.379	0.598–2.832	0.370–4.163	0.319	NS
*MeATTF*	1.679	0.531–4.431	0.384–6.376	0.211	NS
*MeGBF3*	1.094	0.570–1.789	0.272–2.591	0.746	NS
*MeGE3*	1.959	0.501–6.700	0.270–10.729	0.221	NS
*MeGF14*	*2.332*	1.202–5.717	0.824–17.293	0.013	Up-regulation
*MeMYC2*	*1.924*	1.118–3.617	0.741–4.594	0.019	Up-regulation
*MeMSD*	*0.584*	0.429–0.837	0.366–1.282	0.014	Down-regulation
*MeRD28*	1.120	0.990–1.297	0.867–1.409	0.075	NS
*MeP5CS*	1.934	0.746–3.786	0.657–6.197	0.054	NS
*MeZFP*	0.858	0.504–1.471	0.317–2.258	0.539	NS

The relative gene expression levels under drought stress were next compared between the tolerant and susceptible genotypes (Table 5). Under drought stress, 7 of the 10 genes were expressed at significantly higher levels in the tolerant genotype compared with the susceptible genotype. This included four that had been identified as exclusively up-regulated by drought stress in tolerant MH96/0686 (*MeALDH, MeZFP, MeRD28* and *MeMSD*), two that were up-regulated in both genotypes (*MeGBF3, MeATTF*), and one that was exclusively up-regulated in susceptible Nyalanda (*MeP5C5*; however, not up-regulated enough to surpass expression in DT under drought stress). This also included the one gene down-regulated by drought stress in both genotypes (*MeGE3*).

**Table 5:** **Comparison of gene expression levels of DT and DS cassava genotypes after 10 days of stress.** Quantitative RT-PCR was performed for each identified gene on three biological replicates for each genotype (MH96/0686 and Nyalanda) after 10 days of stress. Duplicate reactions were run for every biological replicate. The ΔΔCT method of relative gene quantification was used to make the various comparisons of relative gene expression from the qRT-PCR data, using REST. The DS is used as a calibrator, i.e. the relative expression level of each gene is shown as the relative times higher expression in DT (MH96/0686) than in DS (Nyalanda) under drought stress. The ‘Result’ column indicates whether each gene is significantly expressed at higher levels under drought in MH96/0686 versus Nyalanda. A gene is significantly up-regulated or down-regulated when its expression in a treatment is higher than or lower than in a calibrator (standard/baseline), respectively, and when the *t-*test statistic is lower than 0.05 (at 95 % significant level). The expression in a calibrator is taken as unity (one), expression of more than one is up-regulation and expression less than one is down-regulation. The *t*-statistic will show whether the up-regulation or down-regulation is significant or non-significant (NS).

Gene	Fold higher in MH96/0686 (compared with Nyalanda)	SE	95 % CI	Probability (*P* = 0.05)	Result
*MeALDH*	1.797	1.038–3.258	0.6444.879	0.040	Up-regulation
*MeATTF*	2.040	1.585–2.669	1.377–3.090	0.000	Up-regulation
*MeGBF3*	*1.890*	1.379–2.579	1.099–3.128	0.000	Up-regulation
*MeGE3*	3.033	1.762–5.233	1.122–8.913	0.000	Up-regulation
*MeGF14*	1.330	0.842–1.921	0.669–2.521	0.123	NS
*MeMYC2*	1.180	0.767–1.775	0.634–2.532	0.350	NS
*MeMSD*	1.221	0.800–1.664	0.589–3.109	0.326	NS
*MeRD28*	1.501	1.065–1.966	0.866–2.133	0.017	Up-regulation
*MeP5CS*	1.659	0.992–2.709	0.712–3.197	0.040	Up-regulation
*MeZFP*	2.197	1.115–3.590	0.815–3.900	0.017	Up-regulation

## Discussion

Cassava is an essential crop for increasing food security in SSA and other food-insecure regions. Ranked highest in importance among tropical root crops, its roots are a remarkable food source for 500 million people globally. Subsistence farmers in eastern and central Africa rely heavily on it to survive periods of drought, general crop failure and food scarcity. Although cassava is a relatively DT crop, increased drought tolerance is nevertheless an important trait to consider for improving cassava for resource-poor farmers.

This study conducted morphological, physiological and molecular characterization of a DT improved variety (MH96/0686) and DS landrace (Nyalanda) from Uganda, both identified as such from previous observations in farmers' and research station fields. Characterization of cassava genotypes with disparate responses to drought allowed both characterization of the general cassava drought stress response and identification of the candidate genes that may be contributing to the increased drought tolerance in MH96/0686. These two genotypes represent ideal candidates for integration in the Ugandan cassava breeding programme; the farmer-preferred characteristics of Nyalanda and the drought tolerance and other improved characteristics of MH96/0686 can be combined to produce new varieties combining drought tolerance with high yield under non-drought conditions and other farmer-preferred traits. This study has established molecular tools that can be used to further characterize, understand and breed for drought tolerance in cassava through the inclusion of gene expression-based phenotyping using the drought stress expression-based markers for the identification of quantitative trait loci (QTLs), and finer molecular-level phenotyping of progeny to guide selection of the best genotypes during the breeding programme.

### MH96/0686 resists drought by avoidance while Nyalanda is DS

Physiological and morphological analyses were conducted to assess the drought response of the two genotypes, and confirmed the relative tolerance of MH96/0686 and susceptibility of Nyalanda that had been observed in the field (Turyagyenda *et al*. 2013). Drought stress conditions were designed to more closely reflect those in the field, and those successfully used in other similar studies. Data were collected after 10 days of water stress, when the percentage SMC was close to field capacity (83.00 ± 2.45) in well-watered plants, while in stressed plants it was 28.80 ± 1.08, which is close to the 25 % SMC reported previously as a severe drought stress treatment for cassava (Aina *et al.* 2007). The third fully expanded leaf was beginning to exhibit visual drought stress symptoms in Nyalanda but not in the corresponding leaves in MH96/0686 (Fig. 1), making this leaf a good candidate for assessing the differential response to drought in the two genotypes at a relatively early stage of leaf response.

Measurements indicated that the tolerance of MH96/0686 was due to avoidance at the physiological level (Table 1). The lower stomatal conductance in MH96/0686 compared with Nyalanda is an indication of drought avoidance, reflecting its resistance to water loss through partial stomatal closure for increased water-use efficiency. This represents an effective adaptive response associated with drought tolerance in plants (Heschel and Riginos 2005). Rapid closing of stomata in response to reduced atmospheric humidity and soil moisture has been recognized as the principal mechanism of drought tolerance in cassava (El-Sharkawy 2004; Setter and Fregene 2007). Substantial variation in leaf conductance has been observed and this trait appears to be useful in pre-selecting DT germplasm (Iglesias *et al.* 1995). Lower stomatal conductance is an indication of reduced water loss through stomata for increased water-use efficiency. Wajid *et al.* (2011) also reported that cultivars susceptible to water stress have a higher stomatal conductance and transpiration rate than DT cultivars.

The RWC was significantly higher in MH96/0686 under drought stress (Table 1), a further reflection of drought avoidance achieved by the partial stomatal closure in MH96/0686. Cassava plants that can control leaf loss in response to drought are associated with increased drought tolerance (Lenis *et al*. 2006; El-Sharkawy 2007). As a result of its more limited capacity to cope with drought stress, Nyalanda lost significantly more leaves by shedding and senescence under drought stress (Table 1). Leaf wilting/folding and shedding, as exhibited by Nyalanda, has been described as a drought avoidance mechanism (Mitra 2001) for short-term drought, but this has serious consequences for photosynthesis, whole-plant physiology, productivity under prolonged water stress conditions. Retention of leaves or ‘stay green’ under water stress conditions, as exhibited by MH96/0686, has been correlated with drought tolerance and improved yields in cassava (Lenis *et al.* 2006) because this ‘stay green’ condition maintains photosynthesis. In tobacco, Rivero *et al.* (2007) were able to enhance drought tolerance by delaying drought-induced leaf senescence through transformation using the *isopentenyl transferase* gene. Similarly, the appearance and development of major damage symptoms such as wilting, dying of old leaves and necrosis of young leaves caused by the water stress conditions were delayed in the transgenic rice plants by *HVA1* (group 3 LEA protein) (Xu *et al*. 1996).

Collectively, these data confirm field observations on relative levels of drought tolerance in the two genotypes, indicating that one of the mechanisms that MH96/0686 uses to cope with drought stress is avoidance. These observations support the suitability of these genotypes at this time point for further molecular characterization of drought stress responses in cassava.

### Molecular characterization of cassava drought stress responses and identification of candidate tolerance genes

Other studies have also indicated that a common drought-tolerance mechanism at the physiological level is avoidance, as we have shown is the case for MH96/0686. Therefore, this genotype exhibits a common drought-tolerance mechanism for cassava. These other studies have highlighted the need for molecular analysis of the tolerance response, to help characterize the underlying molecular basis of the tolerance and to provide molecular tools to complement ongoing breeding efforts (El-Sharkawy 2007). Differences in gene expression can serve as expression-based markers for drought stress, and contribute to the drought tolerance of MH96/0686 and of cassava in general. We confirmed that 10 cassava genes are responsive to drought, and further identified the candidate genes underlying the tolerance, which can be used for cassava improvement. The molecular component of this investigation capitalized on the extensive molecular characterization and functional validation studies that have been conducted in other plant species; to optimize the chances of inclusion of functional drought response genes in cassava, a confirmed functional role in drought tolerance in other plant species served as the primary criterion for inclusion in this study.

Molecular characterizations were conducted on the same samples used for morphological and physiological characterizations, from the third fully expanded leaf with 10 days of gradually applied severe drought stress. Unlike recent gene expression studies in cassava by Utsumi *et al.* (2012) that applied a 1-h desiccation shock to identify genes differentially expressed in response to drought, the gradual drought stress used in this study more closely resembles field conditions in order to identify transcriptional changes crucial to adaptation under field conditions (Talame *et al*. 2007). Also, An *et al.* (2012), after subjecting cassava plants to cold stress at 7 °C for different periods, suggested that prolonged stress could trigger more stress-related gene expression. In addition, many molecular studies on drought responses have used a single genotype without comparing the expression of genes between contrasting genotypes (Sakurai *et al*. 2007; Guo *et al.* 2009; You-Zhi *et al*. 2010), making it difficult to separate drought-tolerance-related genes from drought-responsive genes. Genes differentially expressed in response to drought in a single genotype may not necessarily be responsible for enhancing drought tolerance (Guo *et al.* 2009). Contrasting gene expression changes induced by drought stress in tolerant versus susceptible genotypes allow better discrimination of those uniquely responsive in the tolerant genotype, representing candidate genes underlying the tolerance. On the flip side, gene expression changes that are significantly occurring uniquely in the susceptible genotype can serve as markers for drought stress and may contribute to its susceptibility.

When comparing disparately responding genotypes, genes up-regulated in both are less likely to play a significant functional role and are therefore termed ‘drought responsive’, whereas genes up- or down-regulated more significantly in a tolerant genotype represent candidate cassava ‘drought-tolerance’ genes (i.e. may underlie the tolerance of this genotype). Of course, these candidate drought-tolerance genes require further validation to confirm that they play a functional role in tolerance in cassava and specifically in the tolerant genotypes used in this study. Their identification is the first important step and provides insight into the molecular changes in response to drought in the tolerant genotype, and to cassava drought tolerance in general.

### Cassava drought-responsive genes

First, it was assessed whether each of the genes selected from previous studies is responsive to drought in cassava (i.e. whether their expression levels change in response to drought stress). The expression of each of the 10 genes was significantly different when drought stress was applied to either one or both genotypes, compared with well-watered controls. This confirms that all 10 genes are also drought responsive in cassava. They can therefore be used as expression-based markers of drought stress and subjected to further study in the context of drought tolerance of MH96/0686 compared with susceptibility of Nyalanda. The genes commonly up- or down-regulated in both genotypes (*MeGBF3*, *MeATTF* and *MeGE3*; Table 3 and Fig. 2) may constitute part of the general response to drought stress that is commonly invoked within the range of tolerance/susceptibility represented by these two genotypes.

### Candidate drought-tolerance genes

Genes underlying MH96/0686 tolerance, which is no doubt multi-genic, can be divided into two categories: those whose baseline expression levels are different in DT compared with DS (i.e. DT is primed to be less susceptible or responds more quickly to drought stress) and those whose expression levels under drought are significantly more changed/expressed at higher levels in DT compared with DS (i.e. they may play a role in the longer-term tolerance of MH96/0686 observed in the field). The more responsive genes (the second category) represent candidates for the adaptive response of MH96/0686 to drought and therefore important targets for further validation and for subsequent use in developing cassava varieties with enhanced drought tolerance.

In the first category of baseline tolerance genes, two genes were expressed at significantly higher levels in the DT compared with the DS genotype (*MeGF14* and *MeMYC2*) and one at significantly lower levels (*MeMSD*). The second category, consisting of genes with higher expression levels under drought stress in DT compared with DS, included 7 of the 10 genes. The fact that so many of these genes, demonstrated to confer drought tolerance in other species, were up-regulated more significantly by drought in MH96/0686 demonstrates that overall the drought response in this genotype is more robust at a molecular level.

### Genes exclusively up-regulated by drought stress in the DT MH96/0686

Four genes, *MeALDH*, *MeZFP*, *MeRD28* and *MeMSD*, were exclusively up-regulated by water stress in the DT genotype. Under well-watered/control conditions, and using DS as a calibrator, *MeALDH*, *MeZFP* and *MeRD28* were not differentially expressed between DT and DS (Table 4). Using the control treatment as a baseline, this means that the up-regulation of these genes in DT was due to water stress. The expression of *MeMSD* was lower in DT than in DS in the control treatment (well watered) but its expression in DT in response to stress was 2-fold higher than that in DS, indicating that water stress resulted in higher expression of this gene in the DT compared with the DS genotype. Therefore these four genes (*MeALDH*, *MeZFP*, *MeRD28* and *MeMSD*) might be involved in drought adaptation, or the tolerance of MH96/0686 and potentially other tolerant cassava genotypes.

*MeMSD* encodes a manganese superoxide dismutase (MnSOD) enzyme that plays a role in oxidative stress tolerance in plants. Over-expression of superoxide dismutase (SOD) enzymes has been reported to increase oxidative stress tolerance in transgenics (Sen Gupta *et al*. 1993; Van Breusegem *et al*. 1999; Basu *et al*. 2001; Wang *et al*. 2005). For example, Sen Gupta *et al*. (1993) reported that a 3-fold increase in total pea *Cu/ZnSOD* activity resulted in a significant increase in resistance to methyl viologen-induced membrane damage in transgenic tobacco. Basu *et al*. (2001) showed that a 1.5- to 2.5-fold increase in total *SOD* activity in transgenic *Brassica napus* plants transformed with wheat *MnSOD* increased oxidative stress resistance compared with wild-type controls. Wang *et al*. (2005) reported that a 1.4-fold increase in total SOD activity in the *MnSOD* transgenic rice plants was enough to increase oxidative stress resistance and drought tolerance when the gene was fused with a chloroplast transit peptide sequence in order to target the MnSOD to the chloroplast. The 3.148-fold increase observed in DT genotype MH96/0686 therefore indicates a level that can plausibly confer increased oxidative stress and drought tolerance in cassava. Superoxide dismutase enzymes are involved in scavenging reactive oxygen species (ROS) that are produced in plants during water stress (Kendall and McKersie 1989; Price *et al*. 1989; Hernandez *et al*. 1995; McKersie *et al*. 1996; Fryer *et al*. 2002) and so we hypothesize that this gene confers drought tolerance through ROS scavenging in cassava.

The gene *MeZFP* that encodes a zinc finger protein (ZFP252) was also exclusively up-regulated in MH96/0686. This ZFP has been reported to confer drought tolerance in plants by maintaining cell membrane integrity during water stress. Xu *et al*. (2008) reported that the relative electrolyte leakage, an indicator of membrane injury (Morsy *et al*. 2005), was lower in *O. sativa* *ZFP252*-transformed rice plants than in untransformed and *O. sativa* *ZFP252* knock-out plants under drought stress. This suggests that ZFP252 protects plants from stress by maintaining cell membrane integrity. The transformed rice plants also had higher free proline contents and soluble sugars than non-transgenic and *ZFP252* knock-out transgenic rice plants (Xu *et al*. 2008). The findings of Xu *et al*. (2008) suggest that enhanced stress tolerance of *ZFP252*-transgenic plants might partly be through activation of proline synthesis and proline transport pathways by ZFP252 in rice under salt and drought stresses. Proline levels are known to contribute to drought tolerance through osmotic adjustment (Sanchez *et al.* 1998). In this study, *MeZFP* was over-expressed 4.043-fold under drought stress in DT (MH96/0686). It is therefore plausible that this gene is among those that enhance drought tolerance in cassava and specifically in MH96/0686 through increasing the free osmoprotectant proline and soluble sugars.

*MeRD28* encodes the Responsive to Desiccation (*RD28*) gene. The expression of this gene was increased 1.511-fold by water stress, being exclusively upregulated in the DT genotype, suggesting that it plays a role in enhancement of drought tolerance in cassava. Daniels *et al*. (1994) reported that *RD28* is a turgor-responsive, mercury-resistant plasma membrane aquaporin found in plasma membranes of all plant tissues except seeds. Earlier studies by Yamaguchi-Shinozaki *et al*. (1992) reported that RD28 enhances drought tolerance through an abscisic acid-independent pathway. It protects desiccated cells by transporting small molecules across cell membranes and it is believed here that it enhances the cells' desiccation tolerance in DT cassava through osmotic adjustment.

The fourth gene that was exclusively up-regulated in MH96/0686 is *MeALDH*, which encodes aldehyde dehydrogenase (ALDH7B4). It was up-regulated by 2.815-fold under drought stress and may therefore be involved in enhancement of drought tolerance in cassava and specifically MH96/0686. The findings of this study are in agreement with the studies by Kotchoni *et al*. (2006), who reported that transgenic *A. thaliana* plants with increased amounts of ALDH7B4 were more tolerant to dehydration and salt stress than wild-type plants. They further reported that over-expression of the *ALDH7B4* gene in transgenic plants reduced the level of lipid peroxidation under drought and salt stress, suggesting that the gene confers tolerance towards both osmotic and oxidative stress in *A. thaliana* through ROS scavenging and reduction of lipid peroxidation. This gene had also been reported to be induced by pathogens (Zimmermann *et al*. 2004) and therefore might be a multi-stress-responsive gene. Over-expression by 2.815-fold and exclusive up-regulation of this gene in DT cassava are an indication that the gene may be involved in enhancement of drought tolerance in cassava, probably through ROS scavenging and reduced lipid peroxidation.

### Genes up-regulated only in the DS genotype

Three genes (*MeMYC2*, *MeP5CS* and *MeGF14*) were exclusively up-regulated in the DS genotype and not in the DT genotype. These genes are therefore responding to the drought stress state in Nyalanda. However, they are not likely to be part of the genetic basis of drought tolerance in MH96/0686.

### Genes up-regulated by water stress in both DT and DS genotypes

Two genes, *MeATTF* encoding an amino acid transporter family II protein and *MeGBF3* encoding G-box binding factor 3 (GBF3), were up-regulated by stress in both tolerant and susceptible genotypes. They may therefore be among the general drought-responsive genes. GBF3 is one of the several G-box binding factors which are basic leucine zipper (bZIP) proteins (Schindler *et al*. 1992; Izawa *et al*. 1993). In arabidopsis, *GBF3* is highly expressed in roots but not in leaves (Schindler *et al*. 1992) and is believed to be involved in the regulation of alcohol dehydrogenase (*adh*) through an ABA-dependent pathway. The *adh* gene is responsive to cold and dehydration. On the other hand, *MeATTF* has been reported to be up-regulated by water stress (Bray 2004; Guo *et al*. 2009), although its actual function in drought tolerance was not well demonstrated. Although these two genes were up-regulated in both tolerant and susceptible genotypes, their expression in the DT genotype was significantly higher than in the DS genotype (Table 5). Further studies using different genotypes and a large sample size may give insight into whether the two genes are associated with drought tolerance in cassava; however, this study establishes them as expression-based markers of drought stress in cassava that may be useful across a range of genotypes.

### Genes down-regulated by water stress

*MeGE3*, encoding GERMIN 3 (GER3), was down-regulated by water stress treatment in both genotypes. This is in agreement with Bray (2004), who reported that *GER3* (germin-like gene group 3) was repressed by drought stress in leaves of *A. thaliana*. Germin proteins were first identified in wheat as genes that were expressed during germination (Lane *et al*. 1991). The report by Bray (2004) suggested that a possible role for germin-like genes is alteration of cell wall properties that control growth. We speculate that *GER3* down-regulation may contribute to, and is consistent with, the reduction in plant growth observed in both genotypes under drought stress (Table 1).

## Conclusions and forward look

Further characterization of drought tolerance in cassava, especially at the molecular level, is necessary to improve understanding and enhancement of drought tolerance in this plant. The genetic and physiological basis of drought tolerance in cassava was investigated in two cassava genotypes with contrasting tolerance levels to drought stress. The improved, DT variety MH96/0686 exhibited physiological indicators of drought avoidance, through partial or complete closure of stomata to reduce loss of moisture through transpiration. At the molecular level, 10 cassava drought-responsive genes were identified, and further comparisons between the two genotypes helped in the identification of those that are most likely to play a role in drought tolerance in cassava. The genes exclusively up-regulated in DT MH96/0686 represent the most promising candidates for drought-tolerance genes of cassava. Based on these genes' known functions in other plant species, it is likely that MH96/0686 tolerance at the cellular level consists of a reduction of oxidative stress through ROS quenchers (*MeMSD* and *MeALDH*) and osmotic adjustment (*MeZFP* and *MeRD28*). Further research with more genotypes and at more time points, including analysis after re-watering, is warranted to determine whether these and other cassava genes represent drought tolerance QTLs for use in breeding, and for testing their efficacy in conferring drought tolerance on transgenic cassava.

## Sources of funding

This study was supported by funds from the Millennium Science Initiative through the National Council of Science and Technology, Uganda. Supplementary funds were obtained from the National Agricultural Research Organization (NARO), Uganda.

## Contributions by the authors

L.T. conceived and conducted the research, collected the data, conducted experimental design, analysed the data and wrote the manuscript; E.B.K., M.F., Y.B. and D.S.O.O. conceived the research, analysed the data and reviewed the manuscript; M.A. conducted experimental design, analysed the data and reviewed the manuscript; and J.J.W.H. conducted experimental design, analysed the data and co-wrote the manuscript.

## Conflict of interest statement

None declared.
